# Evaluating the Degree of Blending and Properties of Recycled Asphalt Mixtures Containing Fine Reclaimed Asphalt Pavement Particles Designed Across Different Methods

**DOI:** 10.3390/ma19030550

**Published:** 2026-01-30

**Authors:** Dong Liu, Hangcheng He, Yanyan Liu, Haidong Dong, Yining Zhang, Xiaoli Zhan, Mingchen Li, Huailei Cheng

**Affiliations:** 1 Zhejiang Provincial Engineering Research Center for Highway Intelligent Maintenance, Shunchang Highway Maintenance Co., Ltd., Hangzhou 310051, China; zbyikun@163.com (D.L.); lyanyan@zjjtgc.com (Y.L.); dhaidong@zjjtgc.com (H.D.); 2 Zhejiang Key Laboratory of Green Construction and Intelligent Operation & Maintenance for Coastal Infrastructure, Zhejiang University of Technology, Hangzhou 310023, China; 201906080205@zjut.edu.cn (H.H.); zhanxl@zjut.edu.cn (X.Z.); 3 School of Infrastructure Engineering, Dalian University of Technology, Dalian 116024, China; mcli@dlut.edu.cn; 4 The Key Laboratory of Road and Traffic Engineering, Ministry of Education, Tongji University, No. 4800 Cao’an Rd., Shanghai 201804, China; chl6218@126.com

**Keywords:** recycled asphalt mixture, reclaimed asphalt pavement (RAP), degree of blending (DoB), high-modulus asphalt concrete (HMAC), stone mastic asphalt (SMA), performance

## Abstract

Owing to certain inherent deficiencies in their properties, fine reclaimed asphalt pavement (RAP) particles have not yet been widely reused worldwide, resulting in significant environmental pollution and economic waste. Currently, a diverse array of design methods for asphalt mixes has been proposed. These methods can exert a varying influence on the degree of blending (DoB) and the performance of recycled hot-mix asphalt containing fine RAP particles, and some methods may be better suited for recycling fine RAP particles. However, the specific effects and differences among these various methods have yet to be fully revealed. Therefore, this research comprehensively explored these behaviors. Four distinct mix design formulations were investigated: the dense-graded Asphalt Concrete Group (Group AC), the Stone Mastic Asphalt Group (Group SMA), the High-modulus Asphalt Concrete Group (Group HMAC), and the rejuvenator-modified Asphalt Concrete Group (Group AC+Re). It can be found that the DoB and performance varied across different groups. The DoB spanned from 69% to 82%, with Group SMA showing the highest and Group HMAC exhibiting the lowest. The tensile strength ratio (TSR) of Group AC performed only 73.7%, failing to meet the specification threshold; nevertheless, this shortfall can be compensated by employing alternative methods or adding rejuvenator. Group HMAC exhibited the highest splitting-tensile strength and fracture energy. In addition, the incorporation of rejuvenator can enhance most performance of mixes. Some findings may provide a new perspective for the application of fine RAP particles.

## 1. Introduction

Due to multiple benefits such as cost reduction, resource conservation, and environmental protection, reclaimed asphalt pavement (RAP) has been extensively utilized in road construction projects globally for several decades [1,2]. According to a study by Tarsi et al. in 2020, the utilization of RAP materials in the United States can conserve the amount of virgin asphalt binder and aggregates by around 3.1 million tons and 71 million tons, respectively [3]. An additional technical advantage of RAP is its ability to reduce the demand for virgin binder to achieve the optimum binder content (OBC). This is particularly beneficial for incorporating highly binder-absorptive recycled materials, such as recycled concrete aggregate (RCA). The low binder absorption of RAP can effectively offset the high absorption of RCA, facilitating the use of these more challenging recycled materials in asphalt production [4,5]. Moreover, Yao et al. further conducted a comprehensive analysis of the environmental and economic impacts of incorporating RAP into road construction through Life Cycle Cost Analysis (LCCA) and Life Cycle Assessment (LCA), and their study revealed that the inclusion of RAP led to a significant reduction in life cycle costs and greenhouse gas emissions by approximately up to 26.2% and 29.0%, respectively [6]. These deficiencies stem from the aged asphalt binder in RAP, which becomes highly oxidized and enriched in asphaltenes. The chemical structure of these asphaltenes is a key factor; for instance, a recent study on asphaltenes from the Algerian Hassi-Messaoud oil field revealed that insoluble fractions exhibit high oxygen content and uneven compositional structures, which are primary contributors to asphaltene instability and elevated polarity [7]. This fundamental chemical insight helps explain the increased stiffness, brittleness, and poor blending potential commonly associated with aged binders in RAP materials.

Different from the design of traditional virgin asphalt mixtures, the presence of RAP brought a high degree of complexity to the recycled mix design, and numerous studies have indicated that actually these RAP binders were partly activated and blended with virgin binder in the design of recycled hot-mix asphalt (HMA). As early as 2000, based on the frequency sweep test, simple shear test, and indirect tensile test, McDaniel and Anderson firstly pointed out that RAP not just simply acted as “black rock” in recycled asphalt mixtures [8]. Subsequently, some experts and scholars have proposed various methods to quantify the degree of binder activity (DoA) or degree of blending (DoB) between RAP and virgin binder. Huang et al. carried out a mechanical mixing experiment to analyze the quality changes of RAP before and after blending with new materials, and they found that only about 11% of RAP binder was activated and migrated to virgin aggregates during this process [9]. However, this phenomenon only reflected the effect of mechanical friction between the RAP and virgin aggregates. Then, Zhang et al. further proposed a method to quantify DoB in recycled HMAs based on the volumetric parameters, and they found that the DoB basically ranged from 60% to 95% depending on blending duration and RAP source [10]. Moreover, Bowers et al. and Zhao et al. also analyzed the diffusion process of virgin binder into RAP binder during blending using Gel Permeation Chromatography (GPC), Fourier Transform Infrared Spectroscopy (FTIR), and the layered extraction technique. They revealed that the virgin binder diffused into the RAP binder in a gradual manner, beginning with the outer layer, and proposed that the diffusion process basically could be accomplished within 15 min at a mixing temperature of 155 °C for recycled HMA containing 50% RAP; however, the diffusion process required a longer duration when the temperature decreased to 125 °C [11,12].

Recently, some research has explored the design of recycled mixes with different methods. Wang et al. designed recycled warm-mix asphalt with different traditional dense gradations and found that these dense gradations played an important role in the both high- and low-temperature performance of recycled mixes, while they exerted a negligible influence on moisture stability resistance [13]. Devulapalli et al. further designed recycled HMA with stone mastic asphalt (SMA) gradations. The SMA was a gap-graded asphalt mix firstly developed in the 1960s specifically to improve pavement resistance to rutting and to enhance overall durability. They pointed out that incorporation of RAP would reduce the moisture stability of SMA mixes; nonetheless, this adverse effect could be partially offset by the addition of rejuvenating agents to a certain extent [14]. In addition, an Enrobes à Module Élevé (EME) design method for High-Modulus Asphalt Concrete (HMAC) was a specialized asphalt mixture design system developed by French road engineers to meet the demands of heavy traffic and long-lasting pavement performance. The primary goal was to substantially enhance the dynamic modulus of the asphalt mixture through optimized material composition and structural design while also considering fatigue resistance and rutting resistance. Zhu et al. used this EME method to produce high-modulus recycled HMAs. By refining the regeneration process, they found that the improved approach produced recycled mix that was more compact and stable compared to traditional methods; nonetheless, the refined regeneration process may still pose a notable risk for low-temperature cracking [15]. Overall, different design methods may lead to distinct performance characteristics of recycled HMAs. However, current research has not yet fully explored across these different methods.

In the current recycling process, it is encouraged to pre-fractionate RAP materials by sorting them into different size fractions. In 2023, Wang et al. studied the performance of recycled HMA by dividing RAP materials into three particle sizes, including 0–2.36 mm, 2.36–4.75 mm, and 4.75–13.2 mm, prior to utilization and found that this detailed separation process can significantly decrease the variability of results. Antunes et al. also pointed out a similar phenomenon [16]. However, the use of fine particles of RAP materials has been restricted by many governmental agencies due to potential cracking risks in pavement applications. Han et al. found there was a notable decrease in both low-temperature and fatigue performance for the recycled HMAs containing fine RAP particles [17]. Moreover, Zhang et al. found that the recycled HMAs containing fine RAP materials showed poorer moisture stability performance compared to those containing coarse RAP materials [18]. In fact, these fine RAP materials were also rich in RAP content and had remarkable potential environmental and cost benefits. Han et al. found that the recycled HMAs incorporating 50% RAP, with 15% of it being fine particles, could conserve approximately 63% of virgin binder compared to virgin HMAs [17]. More attention may need to be paid to these materials.

While previous studies have independently explored the use of fine RAP [17,18], the effect of DoB [10], and the application of specific mix designs like SMA with RAP [14], a direct and systematic comparison of these design methodologies, especially focusing on the fine RAP particles, is lacking. Crucially, it remains unclear how these different design methods influence the activation and blending efficiency (DoB) of fine RAP, which is rich in aged binder but poses greater performance risks. Therefore, this research not only explores the design of recycled HMA containing fine RAP particles across different methods but also links the choice of design method to the resulting DoB and comprehensive performance. Therefore, this research attempted to explore the design of recycled HMA across different methods and evaluated the DoB and performance of recycled HMA under these methods. The findings of this research may provide a new perspective for the application of fine RAP particles.

## 2. Objectives and Methodology

The objective of this research was to evaluate the degree of blending (DoB) and diverse properties of recycled HMAs containing fine particles of RAP materials, utilizing various design methods. The targeted types of recycled HMAs included traditional dense-graded Asphalt Concrete (AC), Stone Mastic Asphalt (SMA), and High-modulus Asphalt Concrete (HMAC). Additionally, this research also explored the impact of modified rejuvenator (Re) on the performance of recycled HMAs.

**Figure 1:**
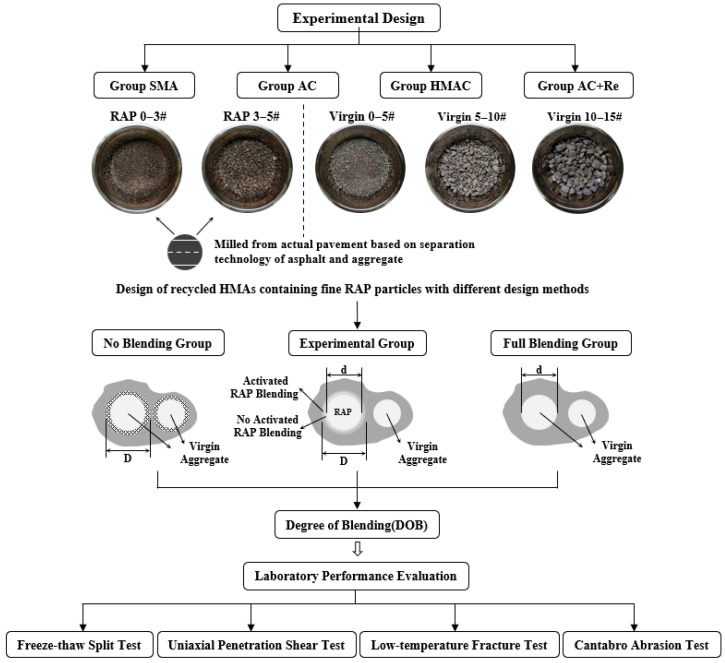
The design flowchart of this research. The D in figure means the diameter of RAP particle, and the d in figure means the diameter of aged aggregate in RAP.

Figure 1 illustrates the design flowchart of this research. Four groups of recycled HMAs containing fine RAP particles were designed with different methods, including Group SMA, Group AC, Group HMAC, and Group AC+Re. To quantify the DoB in each recycled group, four virgin specimen groups were additionally designed and prepared with evenly spaced equivalent asphalt contents. These were intended to simulate the varying degree of blending between RAP binder and virgin binder, encompassing scenarios from no blending to full blending, as well as two intermediate states. The DoB in each group is subsequently determined by comparing the measured volume properties of the recycled HMAs with those of the four calibrated groups. In addition, the performance of these four types of asphalt mixtures has also been evaluated in multiple aspects.

## 3. Material Characterization

### 3.1. RAP Materials

**Figure 2:**
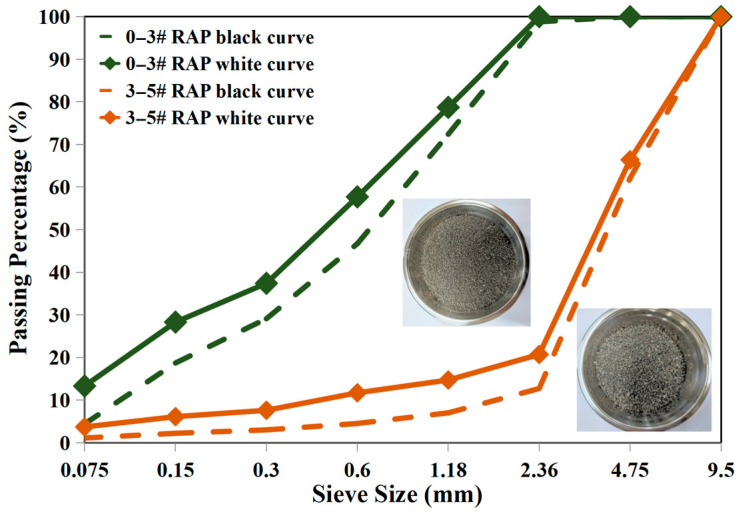
The aggregate gradation of RAP materials black and white curves.

The RAP materials used in this research were milled and recovered from highways in Zhejiang Province, China, that had been in service for over 10 years and were processed using asphalt and aggregate separation technology. This separation technology primarily involved a rotor centrifugal crusher for refined separation and classification. The RAP materials included two size fractions, 0–3# (indicating a particle size range of 0.075–2.36 mm) and 3–5# (indicating a particle size range of 2.36–4.75 mm). A fully automatic extractor and an Absten device were utilized to recover the RAP binder from reclaimed materials. The gradation characteristics of the two fractions of RAP materials before and after extraction are shown in Figure 2. It can be observed that there are significant differences in the gradation of the two RAP materials before and after extraction. The differences in gradations are primarily attributed to the asphalt binder film coating the RAP aggregates and some agglomerations formed by fine particles in the unextracted RAP materials. Additionally, the pertinent properties of RAP binder were measured. The asphalt content, expressed as a percentage by weight of the dry aggregate, for the 0–3# RAP and 3–5# RAP was determined to be 8.5% and 3.4%, respectively. Correspondingly, the penetrations of these two fractions of RAP binders were recorded at 31 (0.1 mm) and 28 (0.1 mm), the softening point were recorded at 56.2 °C and 56.0 °C, and the ductility is 4.6 cm and 5.0 cm, respectively.

### 3.2. Virgin Binder and Rejuvenator

Two types of virgin asphalt binder were utilized in this research, including SBS modified binder and 30 penetration-grade hard binder (abbreviated as 30# hard binder). The SBS modified binder was used for the preparation of specimens in Group SMA, Group AC, and Group AC-Re. Meanwhile, 30# hard binder was employed in the preparation of specimens in Group HMAC and as an equivalent binder for RAP binder in the calibrated groups for DoB assessment. The 30# hard binder, classified by its penetration grade of 20–40 (0.1 mm) according to the Chinese standard (JTG F40-2004) [19], is characterized by its high viscosity and stiffness. It is specifically designed and commonly used in the production of High-Modulus Asphalt Concrete (HMAC) to achieve superior resistance to permanent deformation under heavy traffic loads. The 30# hard binder used in this study was prepared by mixing 70# binder with 16% of hard asphalt particles. The penetration and ductility of SBS modified binder were measured at 43 (0.1 mm) and 41.8 cm, respectively. In addition, the penetration, ductility, and softening point of 30# hard binder were recorded at 34 (0.1 mm), 5.1 cm, and 58.7 °C, respectively. Additionally, the modified rejuvenator was prepared using SBS powder, rejuvenator oil, and a stabilizer through a shearing process, in accordance with the RA-1 type specifications outlined in the technical specification (JTG/T5521-2019) [20].

### 3.3. Virgin Aggregates

In this research, the virgin aggregates were all sourced from limestone, a sedimentary rock primarily composed of the mineral calcite (CaCO_3_). These aggregates are categorized into four types according to their particle sizes: mineral powder, 0–5#, 5–10#, and 10–15#. The passing rates and fundamental physical properties of each aggregate type have been determined and are presented in Table 1. The basic physical properties were determined in accordance with the Chinese standard (JTG E42-2005) [21] for road engineering aggregates. The mechanical characteristics—including the crushing value (19.2–25.1%), water absorption (1.2–1.8%), and density (2.639–2.819 g/cm^3^)—as detailed in Table 1, confirm that the aggregates possess satisfactory strength, low porosity, and are suitable for use in high-performance asphalt mixtures.

## 4. Asphalt Mix Designs

### 4.1. Aggregate Gradation

**Figure 3:**
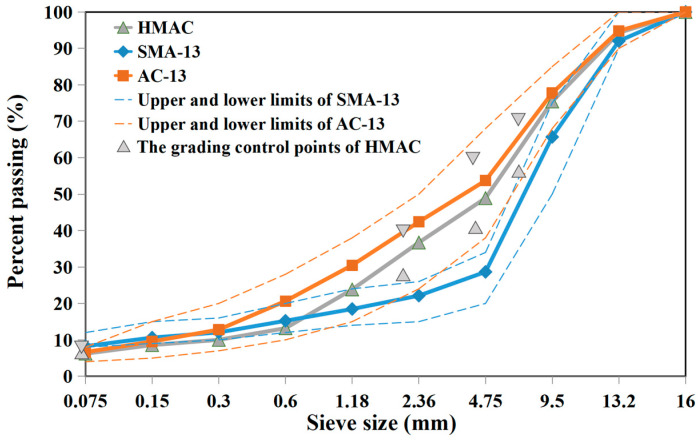
The aggregate gradations adopted in different design methods.

As shown in Figure 3, three different types of aggregate gradations, including AC-13, SMA-13, and HMAC-14, were designed within the specified upper and lower limitations, in accordance with the technical specification (JTG F40-2004) [19] and *LPC Bituminous Mixtures Design Guide* [22]. The RAP gradation used in the design was based on their gradation after extraction. SMA gradation was categorized as discontinuous gradation, characterized by a high content of mineral powder but a relatively low content of fine particles. Conversely, AC gradation was categorized as continuous gradation, which featured a relatively higher content of fine particles. The design of HMAC gradation was based on four pairs of control points, positioning its design range roughly between the SMA and AC gradations.

### 4.2. Mix Design

In this research, for the sake of a more effective comparison, the proportion of RAP materials added in each group was uniformly controlled, with 15% of 0–3# RAP and 5% of 3–5# RAP. This specific ratio was decisively governed by the stringent gradation requirements of the Stone Mastic Asphalt (SMA-13) mix. The 20% total fine RAP content (15% + 5%) was identified as the maximum amount that could be optimally incorporated into the limited fine aggregate portion of the SMA gradation without exceeding its design limits. To ensure a fair comparison across all methodologies, this identical RAP ratio was consistently applied to the AC and HMAC groups. Given that Group SMA required a smaller amount of fine material, the materials within the 0.075 mm to 5 mm particle size range were made up entirely of RAP materials. For the other groups, the material within the 0.075 mm to 5 mm particle size range was a blend of both RAP and virgin materials to different extents. In addition, 0.3% lignin fibers were included into Group SMA, a dosage selected based on common practice and recommendations for SMA mixtures to ensure adequate mortar stability and prevent binder drainage [19]. And a modified rejuvenator was introduced in Group AC+Re, with the rejuvenator content specifically designed to be 5% of the RAP binder. This dosage was determined through preliminary laboratory tests and is aligned with the typical application rates (3–6% of RAP binder) suggested in the technical specification (JTG/T5521-2019) [20] to effectively restore the properties of the aged binder without over-softening the final mixture.

In terms of mix design process, this research utilized the Marshall compactor to prepare specimens. During this procedure, the heating temperature for SBS modified binder and 30# hard asphalt was set at 180 °C, the preheating temperature for RAP material was set at 120 °C, and the heating temperature for virgin materials was 165 °C. The total blending time for Group AC, Group HMAC, and Group AC+Re were all 360 s, with an additional 90 s reserved for Group SMA to mix fibers. The number of compaction blow was consistently designed at 75 blows for all groups. In addition, for Group SMA, Group AC, and Group AC+Re, the optimal asphalt content was determined based on the principle of a 3–5% air void. In contrast, for Group HMAC, the optimal asphalt content was determined by referring to the richness coefficient *K* calculated according to Equation (1) [22]. This is because the French design method for High-Modulus Asphalt Concrete prioritizes the attainment of a high modulus and superior rutting resistance, which is fundamentally achieved by ensuring a rich asphalt content and a very low air void content. In this specification, the value of *K* is required to be greater than 3.4. All the key design parameters for these four groups are presented in Table 2.

(1) $$K=\frac{TL}{\alpha \sqrt[{5}]{\sum }}$$

(2) $$\alpha =\frac{2.65}{G_{se}}$$

(3) 100∑=0.25G+2.3S+12s+135f

where *TL* = the ratio by weight of asphalt to aggregate (%), *G*_*se*_ = the effective specific gravity (g/cm^3^), *G* = the proportion of aggregate particles that are larger than 6.3 mm (%), *S* = the proportion of aggregate particles ranging from 6.3 mm to 0.315 mm, *s* = the proportion of aggregate particles between 0.315 mm and 0.08 mm, *f* = the proportion of aggregate particles smaller than 0.08 mm (%).

### 4.3. Volumetric Properties

Table 3 illustrates the volumetric properties of four groups. Group HMAC exhibited the lowest Voids in the Mineral Aggregate (VMA) value, and with a richness coefficient exceeding 3.4, this group incorporated 3.9% virgin binder, resulting in the lowest air voids of 3.2%. In contrast, Group SMA showed the highest VMA value, and to maintain the 4% air voids, this group added the highest amount of virgin binder. In addition, the property indicators of Group AC and Group AC+Re were intermediate between the aforementioned groups. The VMA and air voids of Group AC+Re were relatively lower than those of Group AC, likely due to the rejuvenator’s effect in enhancing the activation of RAP binder. Overall, the impact of the rejuvenator was not as pronounced as that brought by the gradation.

## 5. Degree of Blending

**Figure 4:**
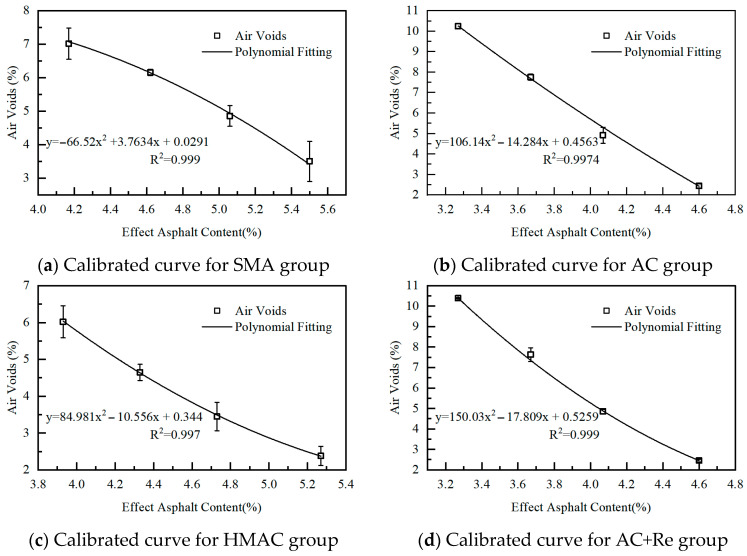
The calibrated blending curves for four recycled groups. y means the air voids; x means the effect asphalt content; R^2^ means the R-squared (Coefficient of Determination).

The DoB between RAP binder and virgin binder was defined as the degree to which RAP binder was activated and mixed with virgin binder or rejuvenator. Since a higher DoB implied that more RAP binder was activated, playing a role in filling air voids and lubricating during compaction, it consequently affected the final volumetric properties of recycled HMAs. Consequently, this research adopted the volumetric parameter-based method introduced by Zhang et al. in 2023 to evaluate the DoB in recycled HMAs [18]. As shown in Figure 4, four calibrated groups of virgin specimens with evenly spaced different equivalent asphalt contents were prepared for each recycled group, encompassing scenarios from no blending state to full blending state, to simulate the various blending degrees in mixes. By polynomial fitting these data, the calibration curves for effective asphalt content (EAC) vs. air voids can be obtained for each group. Then, based on these calibration curves and the measured volumetric results of recycled HMAs as shown in Table 3, the actual EAC in each group can be determined. Here, the EAC was defined as the sum of the virgin asphalt content and the effective RAP binder content. Subsequently, the DoB for each recycled group can be calculated according to Equation (4).

**Figure 5:**
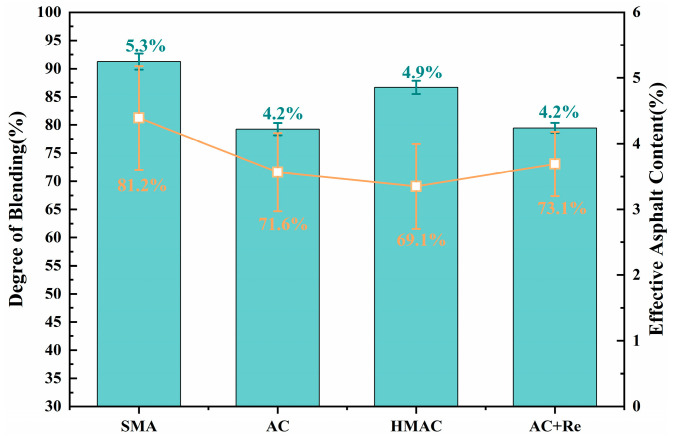
The EAC and DoB for four recycled groups.

Figure 5 illustrates the EAC and DoB results of four recycled groups. A significance analysis of DoB results for each group was conducted using the Tukey pairwise method, with the confidence interval set at 95%, and the significance analysis results are listed in Table 4. This statistical method is widely used following an Analysis of Variance (ANOVA) to identify which specific group means are significantly different from each other while controlling the family-wise error rate. The results of the Tukey test are presented using letter groupings (e.g., A, B, C). Groups that share the same letter are not statistically significantly different from each other at the 95% confidence level (α = 0.05). Conversely, groups that do not share a letter are statistically significantly different. It can be seen that the DoB in recycled HMAs designed through different methods spans an approximate range of 69% to 82%. Group SMA exhibited the highest DoB at 81.2%, significantly higher than the recycled HMAs prepared by other design methods, which may be due to the larger quantity of virgin binder incorporated in this group, resulting in greater contact surfaces for blending. Group HMAC had the lowest DoB value at 69.1%, with a difference of approximately 12% compared to Group SMA. Furthermore, the DoB for Group AC and Group AC+Re was intermediate, around 72%, with the group that included the rejuvenator showing a slightly higher DoB value.

(4) $$DoB\left(\%\right)=\left|\frac{AC_{no\;blending}-EAC_{recycled}}{AC_{no\;blending}-AC_{full\;blending}}\right|\times 100\%$$

where *AC*_*no blending*_ = the asphalt content in calibrated mix designed as no blending state (%); *AC*_*full blending*_ = the asphalt content in calibrated mix designed as full blending state (%); *EAC*_*recycled*_ = the effective asphalt content of actual recycled HMAs.

The volumetric parameter-based method employed in this study provides a practical and effective means to estimate the Degree of Blending (DoB) on a macro-scale, and its correlation with mixture performance has been demonstrated in previous studies [18]. Despite the fact that some micro-scale insights into these blending and diffusion processes cannot be captured, this method can, however, obtain the DoB results in a fast and accurate manner without the need for asphalt extraction from the recycled materials.

## 6. Mixture Performance Testing

**Figure 6:**
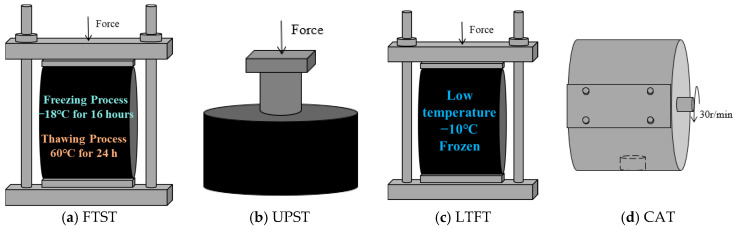
The test devices used to assess the performance of recycled HMAs.

In this research, a thorough series of performance evaluations were conducted on each group of recycled HMAs. The tests included the freeze–thaw splitting test (FTST), uniaxial penetration shear test (UPST), low-temperature fracture test (LTFT), and Cantabro abrasion test (CAT). All these assessments were carried out in strict accordance with the standard test method (JTG/E20-2011) [23]. The test devices are presented in Figure 6. A minimum of four parallel specimens were prepared for each group. The tensile strength ratio (TSR) was employed to assess the water stability of the recycled HMAs. The shear strength (*R*_τ_) served as an indicator of the high-temperature performance. The fracture energy (*G*_f_) and fracture strength were utilized to evaluate the low-temperature resistance of the recycled HMAs. Meanwhile, the percentage loss (Δ*S*) was used to describe the abrasion resistance of the recycled HMAs. These indicators were calculated according to Equations (5)–(9).

(5) $$R_{T_{1}/T_{2}}=0.006287\cdot \frac{P_{T_{1}/T_{2}}}{h}$$

(6) $$TSR=\frac{{\bar{R}}_{T_{2}}}{{\bar{R}}_{T_{1}}}\times 100\%$$

(7) $$R_{\tau }=f_{\tau }\cdot \frac{P}{A}$$

(8) $$G_{f}=\frac{\int_{0}^{D_{f}}f\left(D\right)dD}{h\cdot d}$$

(9) $$\Delta S=\frac{m_{0}-m_{1}}{m_{0}}\times 100\%$$

where *R*_T1/T2_ or *P*_T1/T2_ = the tensile strength or maximum force of specimen before/after freeze–thaw cycle (MPa or N); *h* = the height of specimen (mm); *f*_τ_ = the conversion factor; *A* = the surface area of indenter (mm^2^); *G*_f_ = the fracture work (J); *f*(D) = the load when the vertical displacement equals *D* (kN); Δ*S* = the percentage loss by weight (%); *m*_0_/*m*_1_ = the mass of specimen before or after Cantabro test (g).

## 7. Laboratory Test Results

### 7.1. Freeze–Thaw Splitting Performance

**Figure 7:**
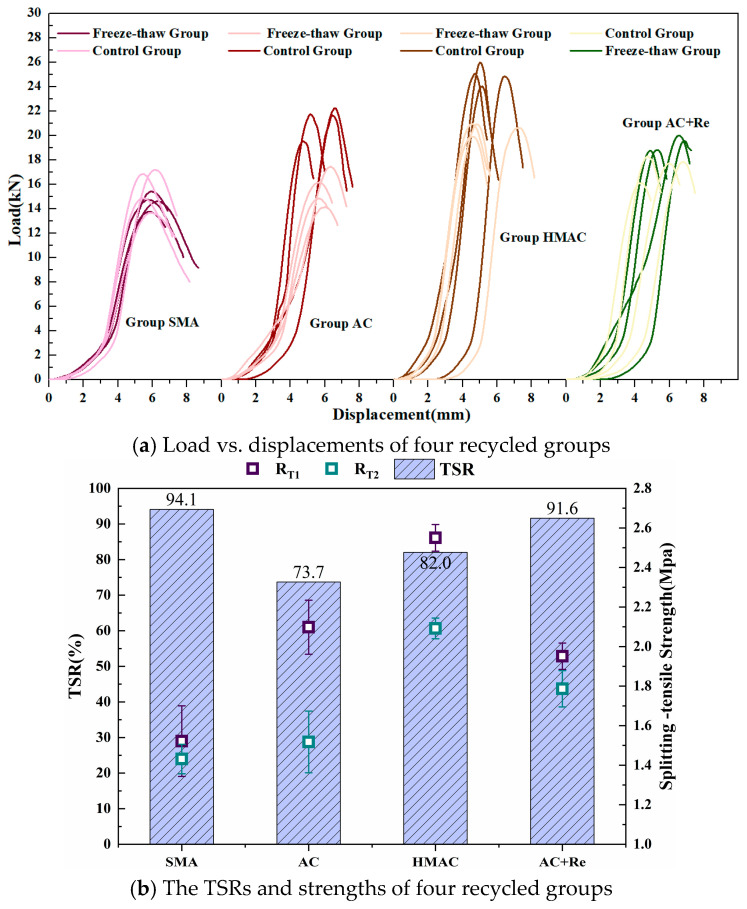
The results of freeze–thaw splitting test.

Figure 7a illustrates the load–displacement curves obtained from the four groups of freeze–thaw splitting tests (FTSTs). Correspondingly, Figure 7b displays the splitting tensile strength results (*RT*_1_ and *RT*_2_) both prior to and following the freeze–thaw cycle, along with the *TSR*. Additionally, a significance analysis was conducted on these indicators using the Tukey pairwise comparison method, employing a 95% confidence interval. The outcomes of this analysis are detailed in Table 5.

The TSR and splitting-tensile strength of recycled HMAs containing fine RAP particles designed through various methods showed significant differences. Group AC, which used a dense-graded design, had the lowest TSR at only 73.7%, failing to meet the specified threshold of 75%, as per the Chinese specification, a shortfall potentially maybe attributable to the inclusion of fine RAP particles. Nevertheless, the addition of a modified rejuvenator markedly enhanced the TSR performance of recycled HMAs containing fine RAP particles. Here, Group AC+Re showed a significant increase in TSR by approximately 17.9% compared to Group AC. In addition, Group SMA exhibited the highest TSR at 94.1%, likely due to its higher effective asphalt content, although its splitting-tensile strength was relatively lower. In contrast, Group HMAC showed the highest splitting tensile strength, nearly double that of Group SMA, with its TSR at approximately 82%, positioning it between Group AC and Group SMA. Therefore, compared to dense-graded design, using other design approaches or incorporating modified rejuvenators may be more advantageous for the water stability of recycled HMA containing fine RAP particles.

### 7.2. High-Temperature Performance

**Figure 8:**
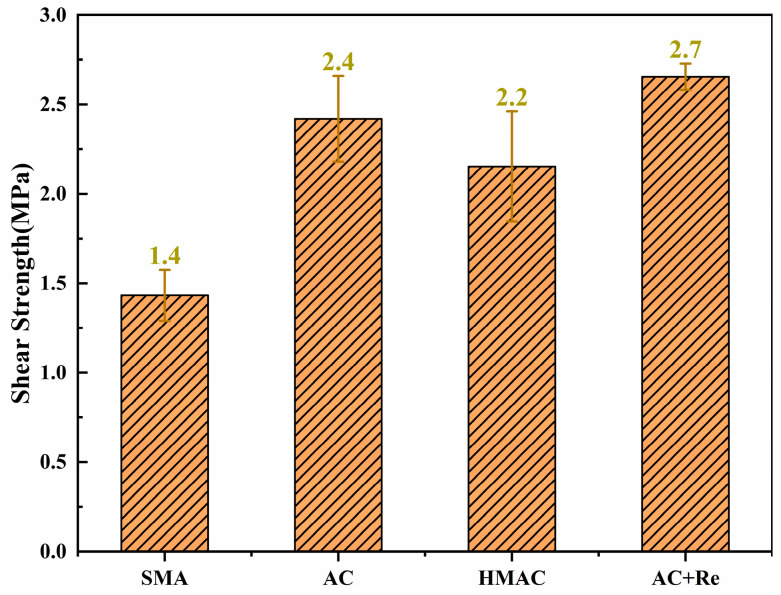
The results of high-temperature performance test.

The results of shear strength are illustrated in Figure 8. A significance analysis was conducted on the indicator using the Tukey pairwise comparison method, employing a 95% confidence interval, as shown in Table 6.

It can be observed that Group AC in this research showed a high shear strength of 2.4 MPa. The addition of modified rejuvenator may further facilitate the blending at the interface between RAP and virgin binder, thereby enhancing the shear strength of recycled HMAs. Specifically, here, Group AC+Re exhibited a 10% increase in shear strength compared to Group AC, with an improvement of 0.2 MPa. In addition, due to the increased asphalt content and reduced air voids, Group HMAC showed a shear strength of 2.2 MPa, which was slightly lower than that of the former groups, yet there was no significant statistical difference when compared to Group AC. Moreover, Group SMA showed the lowest shear strength among the groups though still relatively high at 1.4 MPa. Traditionally, virgin HMAs designed using the SMA method tended to have superior high-temperature performance compared to those designed using the AC method. However, in this instance, the inclusion of fine RAP particles and the use of SBS modified binder in both groups allowed them to possess adequate shear strength. Moreover, likely a result of the gradation characteristics of Group SMA that necessitated 0.91% more asphalt content in its design compared to Group AC, the shear strength of Group SMA was relatively lower.

### 7.3. Low-Temperature Performance

**Figure 9:**
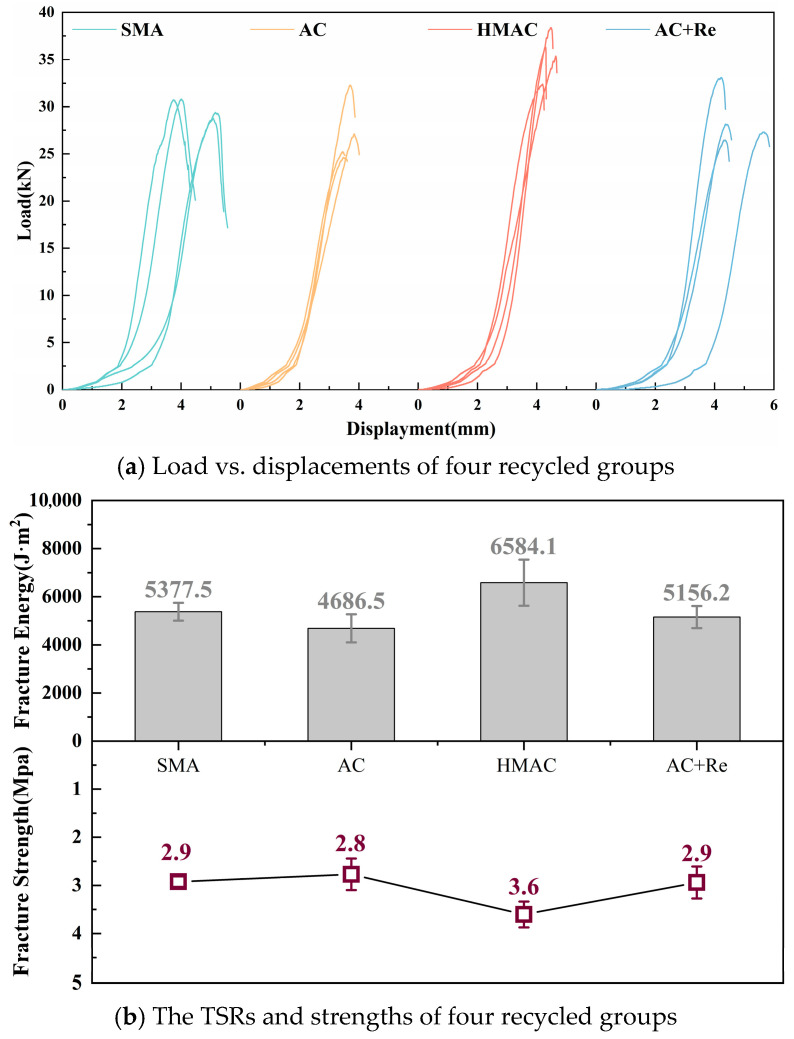
The results of low-temperature performance test.

The load–displacement curves for four groups of LTFT are depicted in Figure 9a, and the results of fracture energy and fracture strength are illustrated in Figure 9b. A significance analysis of these indicators was conducted using the Tukey pairwise method, and the significance analysis results are listed in Table 7.

In this research, the fracture energy and fracture strength of recycled HMA containing fine RAP particles designed using different methods showed significant differences. Group HMAC showed the highest fracture energy and fracture strength, reaching 6584.1 J·m^2^ and 3.6 MPa, respectively, followed by Group SMA. In contrast, Group AC showed the lowest fracture energy and fracture strength, at 4686.5 J·m^2^ and 2.8 MPa, respectively. Here, despite using a low penetration grade of asphalt binder, Group HMAC still presented a superior low-temperature cracking resistance, which can be attributed to its thicker asphalt film and reduced air voids in the design. Group SMA, with its higher asphalt content, also exhibited better low-temperature performance. Moreover, it can be found that the incorporation of a modified rejuvenator can also contribute to enhancing the low-temperature performance of the recycled HMAs to a certain extent. In this instance, Group AC+Re showed an increase of 469.7 J·m^2^ (10%) and 0.2 MPa (6%) in fracture energy and fracture strength, respectively, compared to Group AC.

### 7.4. Cantanbro Abrasion Performance

**Figure 10:**
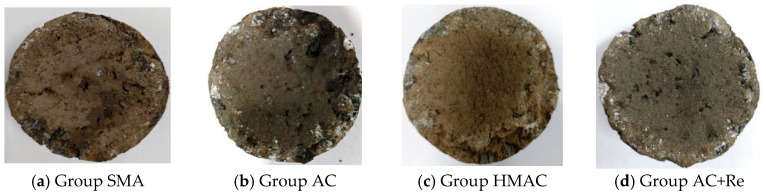
Shapes of specimens after 300 revolutions in CAT.

**Figure 11:**
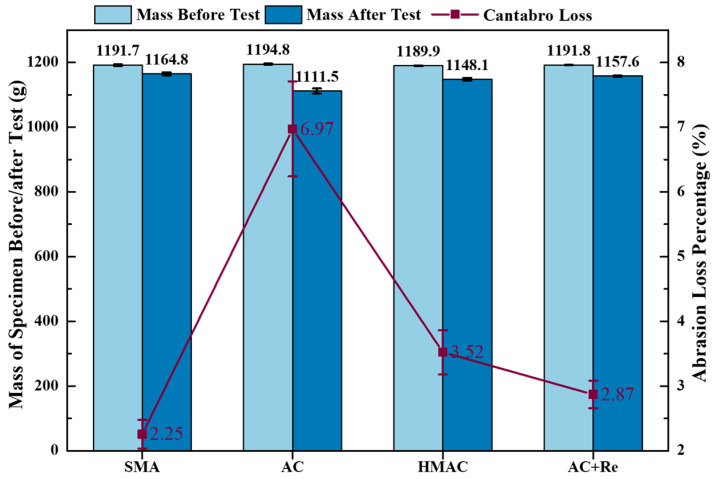
The results of Cantabro abrasion performance test.

The condition of the recycled specimens after 300 revolutions in the Cantabro abrasion test are presented in Figure 10, and the results of abrasion loss percentage are illustrated in Figure 11. A significance analysis of these indicators was conducted using the Tukey pairwise method, and the significance analysis results are listed in Table 8.

It can be seen that the abrasion loss percentage of recycled HMAs containing fine RAP particles designed using different methods all fell within the specification threshold requirement of 15%. However, there were notable differences in the abrasion results obtained by different methods. Maybe due to the higher EAC and DoB, Group SMA showed the most exceptional abrasion performance, with only 2.3% in abrasion loss percentage. In contrast, Group AC showed the worst abrasion performance, with an abrasion loss percentage of 7.0%. The addition of modified rejuvenator can significantly enhance the abrasion performance of the recycled HMAs containing fine RAP particles. Here, the abrasion loss percentage of Group AC+Re reached 2.9%, a reduction of 4.1% compared to Group AC. In addition, in this research, the abrasion loss percentage of Group HMAC was 3.5%, and the value was between Group AC and Group SMA. This phenomenon may be caused by the combined effects of the gradation and EAC of the three groups of recycled materials.

## 8. Summary and Conclusions

This research explored the effect of designing recycled HMA containing fine RAP particles across different methods and evaluated the DoB and performance of recycled HMA under these methods. Four types of recycled HMAs were designed, including Group SMA, Group AC, Group HMAC, and Group AC+Re. Based on the findings, the following conclusions can be drawn:

(1) The DoB in recycled HMAs containing fine RAP particles across different methods spans an approximate range of 69% to 82%. Group SMA exhibited the highest DoB, significantly higher than the recycled HMAs prepared by other design methods, which may be due to the larger quantity of virgin binder incorporated in this group. Group HMAC had the lowest DoB value at 69.1%. The DoB in Group AC and Group AC+Re was intermediate, around 72%, with the group including the rejuvenator showing a slightly higher DoB value.

(2) The performance in recycled HMAs designed across different methods also showed significant differences. The TSR of Group AC showed the lowest level of only 73.7%, failing to meet the specification threshold. Nevertheless, this shortfall can be well compensated by employing alternative design methods or adding a modified rejuvenator. Group HMAC exhibited the highest splitting-tensile strength and fracture energy among groups, likely due to the addition of hard binder and the design with a thicker asphalt film. In addition, the incorporation of modified rejuvenator can simultaneously enhance the DoB and almost all performance of recycled HMAs containing fine RAP particles.

(3) In addition, Group SMA showed the most exceptional abrasion performance, with only 2.3% in abrasion loss percentage, and in contrast, Group AC showed the worst abrasion performance, with an abrasion loss percentage of 7.0%. However, the situation was different in the high-temperature performance, Group SMA showed the lowest shear strength among the groups, though still relatively high at 1.4 MPa, and Group AC and Group AC+Re showed the highest value of shear strength.

(4) The concurrent enhancement of the Degree of Blending (DoB) and key performance properties (moisture stability, low-temperature cracking resistance, abrasion resistance) in Group AC+Re can be attributed to the fundamental role of the rejuvenator in re-activating the aged RAP binder. Mechanistically, the rejuvenator diffuses into the hardened RAP binder, replenishes the lost maltene fractions, and effectively reduces its viscosity and stiffness. This process increases the molecular mobility of the aged binder, thereby promoting its liberation from the RAP aggregate surface and improving its blending efficiency with the virgin binder (leading to a higher DoB). Simultaneously, this restoration of binder properties enhances the cohesion of the composite binder system, which directly translates to the observed improvements in performance.

(5) This study demonstrates that the performance limitations of fine RAP are not inherent but can be strategically engineered out through informed mix design. The findings provide a performance-based selection framework: SMA is recommended for superior moisture stability and durability, HMAC for applications demanding high stiffness and fracture resistance, and AC modified with a rejuvenator as a versatile and effective solution. This work validates a design-for-recycling approach, enabling a significant increase in fine RAP utilization. By transforming this challenging material into a reliable resource, the pavement industry can achieve substantial reductions in virgin material consumption, lower costs, and advance towards more sustainable construction practices.

## Figures and Tables

**Table 1:** The passing rate and basic properties of virgin aggregates.

Sieve Size	0.075	0.15	0.3	0.6	1.18	2.36	4.75	9.5	13.2	16.0
10–15#	0.1	0.1	0.1	0.1	0.1	0.1	0.4	13.0	82.0	100.0
5–10#	0.2	0.2	0.2	0.2	0.2	0.4	6.8	94.9	100.0	100.0
0–5#	1.5	3.5	10.0	26.6	50.2	79.7	100.0	100.0	100.0	100.0
Mineral	100.0	100.0	100.0	100.0	100.0	100.0	100.0	100.0	100.0	100.0
Physical Properties	Crushing Stone Value (%)				Water Absorption (%)			Density (g/cm^3^)		
	25.1% (for 0–5#)				1.5% (for 0–5#)			2.639 (for 0–5#)		
	22.4% (for 5–10#)				1.8% (for 5–10#)			2.744 (for 5–10#)		
	19.2% (for 10–15#)				1.2% (for 10–15#)			2.819 (for 10–15#)		

**Table 2:** The design parameters of four groups designed in this research.

Groups	RAP Percentage (%)		Virgin Materials Percentage (%)				Binder Type	Additions	Asphalt Content (%)	
	0–3#	3–5#	Mineral	0–5#	5–10#	10–15#			Virgin	RAP
Group SMA	15.0	5.0	6.0	0.0	37.0	37.0	SBS	Lignin Fiber	4.2	1.3
Group AC	15.0	5.0	4.0	28.0	24.0	24.0	SBS	/	3.3	1.3
Group HMAC	15.0	5.0	4.0	23.0	26.5	26.5	30#	/	3.9	1.3
Group AC+Re	15.0	5.0	4.0	28.0	24.0	24.0	SBS	Rejuvenator	3.3	1.3

**Table 3:** The volumetric properties of four groups designed in this research.

Groups	Density (g/cm^3^)	Air Voids (%)	VMA (%)	VFA (%)
Group SMA	2.385	4.3	16.0	73.1
Group AC	2.427	4.3	13.8	69.3
Group HMAC	2.435	3.2	9.6	66.8
Group AC+Re	2.431	4.1	13.6	70.2

**Table 4:** Tukey pairwise analysis of DoB results at α = 0.05.

Factor	Grouping	
	DoB	
Group SMA	A	
Group AC+Re		B
Group AC		B
Group HMAC		B

Note: groups that do not share a letter are significantly different.

**Table 5:** Tukey pairwise analysis of FTST results at α = 0.05.

Factor	Grouping				Factor	Grouping			Factor	Grouping	
	R_T1_					R_T2_				TSR	
Group HMAC	A				Group HMAC	A			Group SMA	A	
Group AC		B			Group AC+Re		B		Group AC+Re	A	
Group AC+Re			C		Group AC		B	C	Group HMAC		B
Group SMA				D	Group SMA			C	Group AC		B

Note: groups with different letters indicate significant differences.

**Table 6:** Tukey pairwise analysis of UPST results at α = 0.05.

Factor	Grouping		
	Shear Strength		
Group AC+Re	A		
Group AC	A	B	
Group HMAC		B	
Group SMA			C

Note: groups with different letters indicate significant differences.

**Table 7:** Tukey pairwise analysis of LTFT results at α = 0.05.

Factor	Grouping		Factor	Grouping		
	Fracture Energy			Thermal Fracture Strength		
Group HMAC	A		Group HMAC	A		
Group SMA	A	B	Group AC+Re		B	C
Group AC+Re	A	B	Group SMA		B	
Group AC		B	Group AC			C

Note: groups with different letters indicate significant differences.

**Table 8:** Tukey pairwise analysis of CAT results at α = 0.05.

Factor	Grouping			
	Abrasion Loss Percentage			
Group SMA	A			
Group AC+Re		B		
Group HMAC			C	
Group AC				D

Note: groups with different letters indicate significant differences.

## Data Availability

The original contributions presented in this study are included in the article. Further inquiries can be directed to the corresponding author.
